# Novel Homozygous Consensus Splice Site Variant in the BORCS8 Gene Associated With Fetal Semi-lobar Holoprosencephaly

**DOI:** 10.7759/cureus.116976

**Published:** 2026-09-26

**Authors:** Parag M Tamhankar, Vasundhara Tamhankar, Deval Shah, Tushar Kachhadiya, Shilpa Mithbawkar, Shalin Vaniawala, Jyoti C Suvarna, Salil Vaniawala, Pramila Menon, Shailaja Mane, Dhavendra Kumar

**Affiliations:** 1 Pediatrics, Dr. D. Y. Patil Medical College, Hospital and Research Centre, Dr. D. Y. Patil Vidyapeeth (Deemed to be University), Pimpri, Pune, IND; 2 Genetics, Centre for Medical Genetics, Mumbai, IND; 3 Genetics, SN Gene Lab Pvt Ltd, Surat, IND; 4 Obstetrics and Gynaecology, Astha Hospital, Deesa, IND; 5 Pediatrics, Holy Spirit Hospital, Mumbai, IND; 6 Genetics, The William Harvey Research Institute, Barts and The London School of Medicine and Dentistry, Queen Mary University of London, London, GBR; 7 Apollo Genetics Institutes, Apollo Hospitals, New Delhi, IND

**Keywords:** exome sequencing, holoprosencephaly(hpe), materno fetal medicine, novel mutation, sanger sequence, snp microarray, splice site mutation

## Abstract

Holoprosencephaly (HPE) is a structural malformation of the brain resulting from incomplete cleavage of the forebrain (prosencephalon) into right and left hemispheres, deep brain structures, olfactory bulbs, optic bulbs, and tracts. Mutations in any of the following genes, *C2CD3, CDON, CNOT1, CENPF, DHCR7, DISP1, DLL1, EMX2, FGF8, FGFR1, GLI2, GLI3, ICK, IFT80, KMT2D, PPP1R12A, PRRX1, PTCH1, RAD21, SHH, SIX3, SMC1A, STAG2, STIL, TGIF1, WDR81, *and* ZIC2,* can lead to HPE. Herein, we report for the first time, to the best of our knowledge, recurrent fetal semi-lobar HPE in association with a homozygous pathogenic consensus splice site variant in the BLOC-1-related complex subunit 8 (*BORCS8*) gene (c.150+1G>T, NM_001145784.2) or (chr19-19186892 C>A, GRCh38 format), identified on exome sequencing. Chromosomal microarray results were normal. However, the gene *BORCS8 (MEF2B)* was found in a region of loss of heterozygosity arr[hg19] 19p13.2p12(13,017,875-20,684,670) hmz (around 7667 kilobases), which suggested identity of this region by descent (result of consanguinity). Parental Sanger sequencing confirmed them as asymptomatic carriers (heterozygous) for the same variant (autosomal recessive inheritance). The *BORCS8* gene encodes a protein subunit of the BLOC-one-related complex (BORC) in lysosomes that helps them to be transported to the peripheral cytoplasm in non-neuronal cells and the distal axon in neurons. Homozygous loss-of-function variants in the *BORCS8* gene have been recently shown to lead to severe early childhood-onset neurodegenerative disorder. Clinical features described in these cases were cerebral and cerebellar atrophy, hypomyelination, and a thin corpus callosum. However, fetal presentation of HPE has not been described in the literature to date. We postulate that this could be due to a homozygous amorphic (complete loss-of-function) variant in our case, compared to the homozygous hypomorphs (partial loss-of-function) or compound heterozygous amorphic-hypomorphic variants described in the previously published childhood-onset cases. This case report expands the phenotypic spectrum of the *BORCS8* gene-related disease. The *BORCS8* gene may thus represent a candidate gene for HPE and further studies or reports of similar cases are needed to confirm or refute a causative association. This is the third published paper related to the *BORCS8* gene-related disease as per the PubMed database (MEDLINE).

## Introduction

Holoprosencephaly (HPE) is a developmental anomaly of the brain wherein there is a failure of division of the prosencephalon (forebrain) into two hemispheres during the embryonic period. The phenotypic spectrum varies from alobar HPE (most severe) to lobar HPE (least severe). Alobar HPE features a complete lack of separation of the two hemispheres with a central monoventricle. Facial features can include cyclopia (single eye), nose replaced by a proboscis above the eye, anophthalmia/microphthalmia, and median cleft lip/palate. Middle interhemispheric variant (MIHV) shows partial cleavage of the brain anteriorly and posteriorly but variable fusion of the “middle structures” such as the posterior part of the frontal lobe, parietal lobe, thalami, and basal ganglia. Facial appearance can be normal, or subtle features, such as closely spaced eyes and a depressed nasal bridge, may be present. Semi-lobar HPE features fusion of the frontal lobes and parietal lobes, leading to an anterior monoventricle, but the presence of posterior cleavage (occipital lobes) to form posterior horns of the lateral ventricle. Facial features can include ocular hypotelorism, anophthalmia or microphthalmia, and cleft lip/palate. Lobar HPE features almost complete division of the forebrain, except for the most rostral and ventral parts. It’s the mildest form, with facial features being mostly normal, but ocular hypotelorism, a single central incisor, and cleft lip/palate may be present occasionally [1,2]. 

The causes of HPE can include environmental factors (teratogens), chromosomal aberrations, and single-gene diseases. Maternal diabetes mellitus increases the risk of HPE more than 10-fold. Chromosomal aberrations occur in 25% to 50% of cases, with Trisomy 13 being the most common cause. In addition, several genes have been identified that can cause HPE. The causative genes include *C2CD3, CDON, CNOT1, CENPF, DHCR7, DISP1, DLL1, EMX2, FGF8, FGFR1, GLI2, GLI3, ICK, IFT80, KMT2D, PPP1R12A, PRRX1, PTCH1, RAD21, SHH, SIX3, SMC1A, STAG2, STIL, TGIF1, WDR81, *and* ZIC2 *[1,2]. The most frequently involved genes include *SHH, ZIC2, SIX3,* and *TGIF1*. The genetics of HPE involve a complex network of signaling pathways, primarily the Sonic Hedgehog (SHH), Nodal, and FGF pathways. While some genes cause "nonsyndromic" HPE (where the brain malformation is the primary finding), many others cause broader multisystem syndromes where HPE is only an occasional feature. Many of these genes, viz. *SHH, PTCH1, DISP1, GLI2, *and *CDON*, interact within the *SHH* pathway, which is essential for ventralizing the forebrain. The majority of nonsyndromic HPE genes, such as *SHH, ZIC2, *and* SIX3*, follow an autosomal dominant (AD) pattern with incomplete penetrance. Autosomal recessive (AR) inheritance is seen in syndromic forms with variants in genes such as *DHCR7* or *STIL* [1,2].

Clinically, HPE is associated with significant morbidity, including severe developmental delay, difficult-to-control seizures, and hypothalamic or pituitary dysfunction, most commonly manifesting as central diabetes insipidus. Feeding difficulties, instability of vital signs, and hydrocephalus are also frequent complications. While more severely affected infants with cyclopia may not survive beyond the neonatal period, a significant proportion of children with milder forms, and rarely cases of alobar HPE, survive into adulthood. However, almost all survivors remain fully dependent on caregivers. Evaluation of a proband requires a multidisciplinary approach, starting with high-resolution cranial MRI to define the subtype, followed by a detailed physical examination of both the child and parents to identify subtle microforms. Genetic testing typically begins with a chromosomal microarray (CMA) to detect copy number variations (CNVs), followed by multigene panels or exome sequencing if initial tests are negative. Genetic counseling is vital, as the extreme variability of expression means that a parent with a nearly imperceptible microform carries a 50% risk of passing the variant to offspring who may be more severely affected [1]. 

We report for the first time (to the best of our knowledge) a case of antenatally detected semi-lobar holoprosencephaly in association with a homozygous pathogenic consensus splice site variant in the BLOC-1 related complex subunit 8(*BORCS8*)* *gene (c.150+1G>T or chr19-19186892 C>A, transcript ID NM_001145784.2), identified on exome sequencing. The *BORCS8* gene encodes BLOC-1-related complex subunit 8, a component of the BORC complex involved in endosome and lysosome size regulation, lysosome localization, and organelle transport along microtubules. Homozygous loss-of-function variants in the *BORCS8* gene have been recently shown to lead to severe early childhood-onset neurodegenerative disorder [3,4]. However, fetal presentation of holoprosencephaly has not been described in the literature or in current databases such as Online Mendelian Inheritance in Man (OMIM) or PubMed (MEDLINE) to date [5,6]. This case report expands the phenotypic spectrum of *BORCS8* gene-related disease. The *BORCS8* gene may represent a candidate HPE gene, and additional independent case reports are required to formally include it on HPE diagnostic gene panels. We also propose possible mechanisms by which a pathogenic variant in the *BORCS8* gene could lead to HPE in humans.

## Case presentation

A third-degree consanguineous couple presented with natural conception in their late second trimester. The mother did not receive periconceptional folate. There was no history of maternal diabetes, pregnancy-induced hypertension, rash, or viral infections during pregnancy, and no intake of teratogenic drugs, alcohol, or smoking. At around 26 weeks, an anomaly scan was performed. The scan showed a singleton live fetus with mean overall growth at the 19th centile. The liquor was adequate. The scan showed the following biometry: biparietal diameter 6.5 cm (26 weeks 1 day), head circumference 24.42 cm (25 weeks 4 days), occipital- frontal diameter 8.5 cm, abdominal circumference 20.82 cm (25 weeks 1 day), femur length 4.81 cm (26 weeks 1 days), humerus length 4.4 cm (26 weeks 1 days), trans-cerebellar diameter 2.43 cm (22 weeks 2 days, less than 1st centile for 26 weeks); transverse diameter of the cisterna magna was 6.76 mm. The scan of the fetal skull showed a semilobar type of holoprosencephaly: fused frontal horns and bodies of both lateral ventricles, occipital horns were separated, and thalami were cleaved; the midline falx was absent anteriorly and present in the occipital region. The cerebellum was below the 1st centile with absent vermis (fused two hemispheres), suggestive of rhombencephalosynapsis (Figures 1-4).

**Figure 1:**
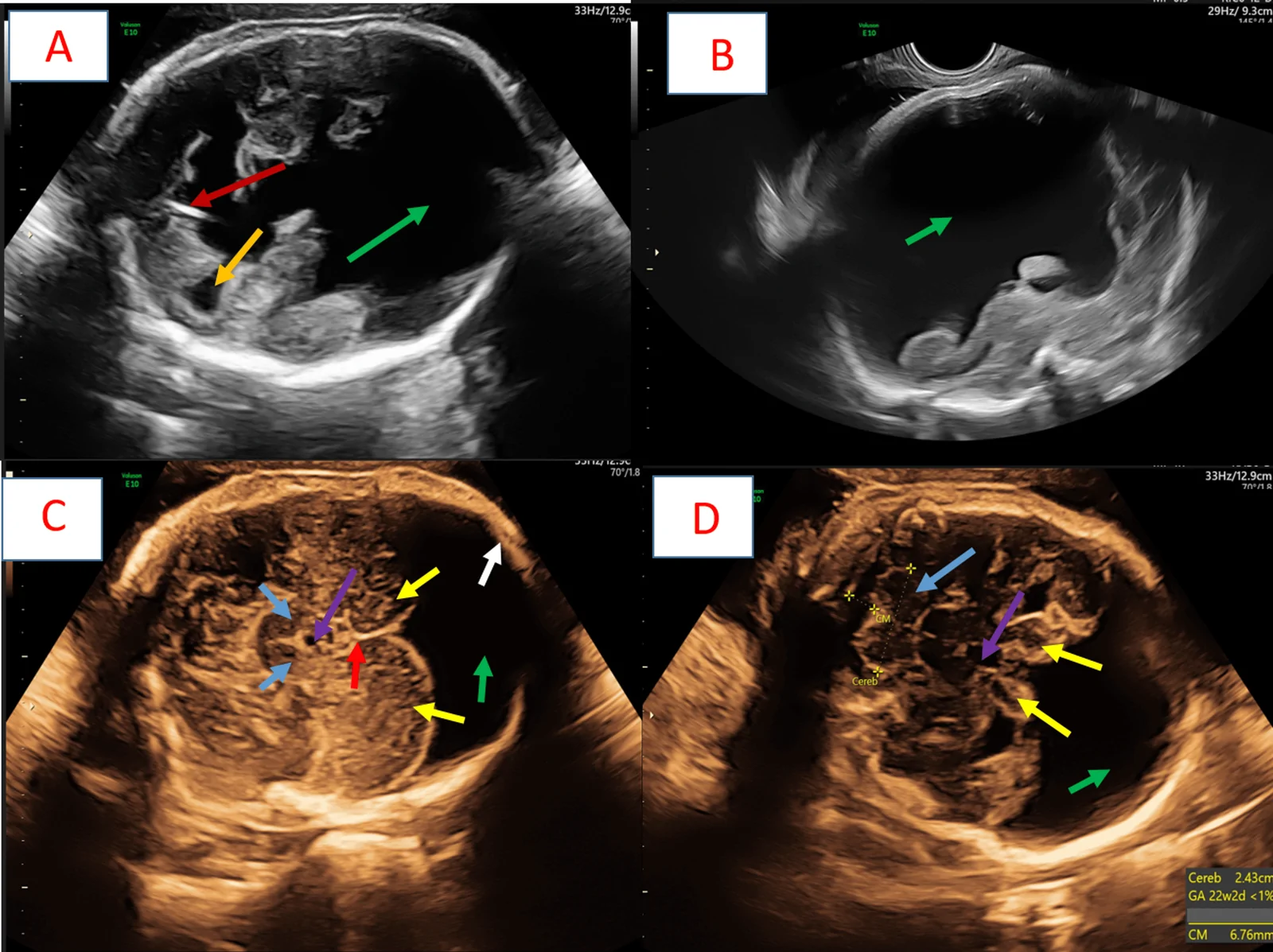
Ultrasound image of the fetal brain with semilobar holoprosencephaly in the couple's first pregnancy Ultrasound image of the fetal brain with semilobar holoprosencephaly in the couple's first pregnancy, showing A) a transverse plane passing at the level of the frontal horns of the lateral ventricles. B) In the sagittal section, one can note severe disruption of the midline architecture, lack of a normal corpus callosum, and presence of a monoventricle anteriorly (green arrow). C) In this transverse section at level of the thalami the image shows a monoventricle on the right (green arrow) made up of fused anterior horns and central part of the lateral ventricles, the thalami are seen fused (blue arrows) with a small rudimentary third ventricle (purple arrow), the frontal lobes (yellow arrows) are fused with absent cavum septum pellucidum (red arrow). The anterior thin cerebral mantle (white arrow) can also be seen as a layer of cerebral tissue stretched over the fluid. D) A transverse plane passing through the partially fused thalami (yellow arrows), third ventricle (purple arrow), and the cerebellum (cereb) (blue arrow). The cerebellum is hypoplastic; the trans-cerebellar diameter is 2.43 cm, corresponding to 22 weeks 2 days, which is less than the 1st centile for 26 weeks (normal is 2.7 cm to 3 cm), the vermis is absent, and the hemispheres are fused, suggestive of cerebellar rhombencephalosynapsis. Cisterna magna (CM) is 6.76 mm (normal is 2 - 10 mm). The fetus is 26 weeks by the last menstrual period.

**Figure 2:**
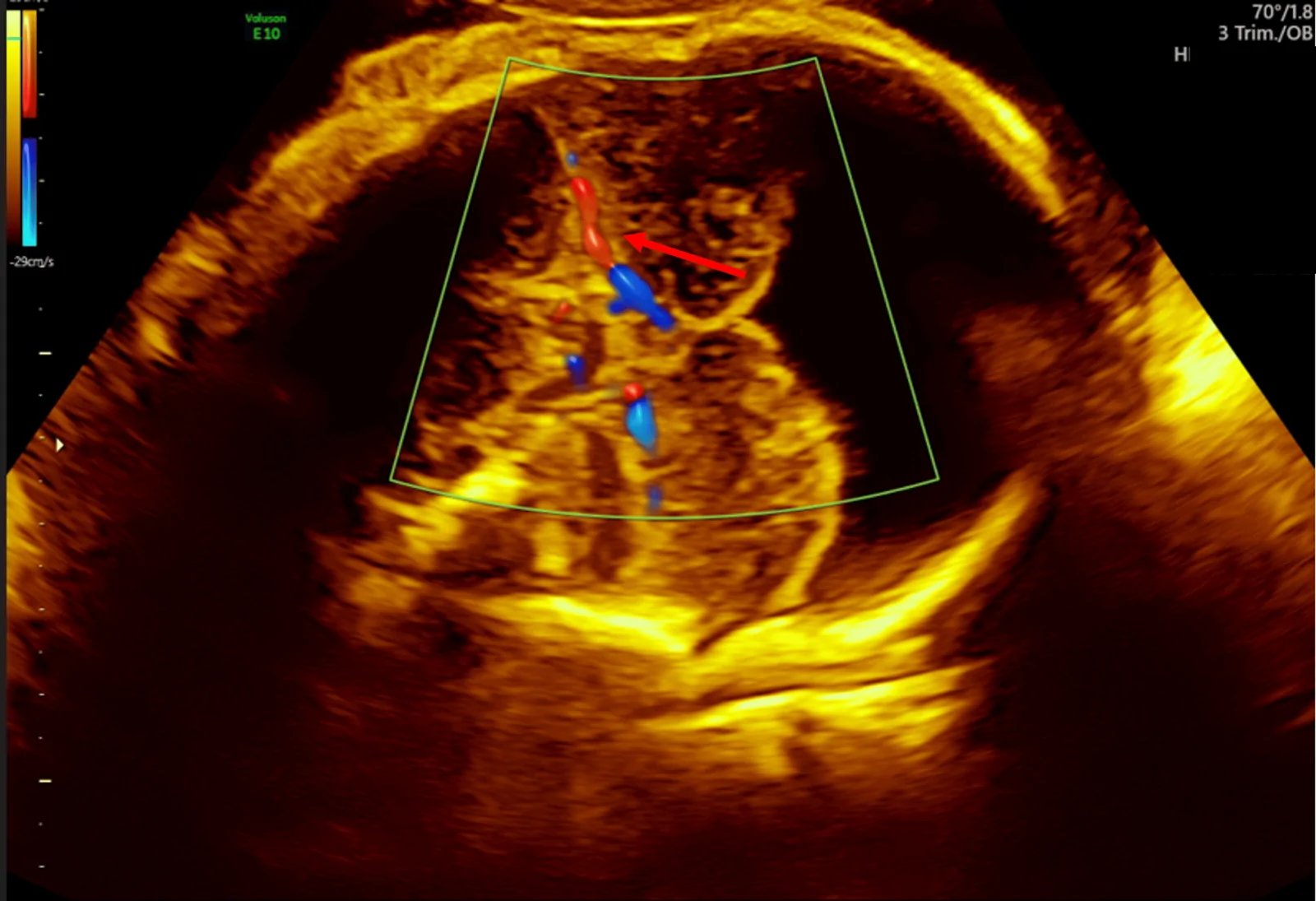
Color Doppler of the fetal brain (transverse plane at level of thalamus) in the first pregnancy Color Doppler image of the fetal brain shows the displacement of the cerebral vasculature. The anterior cerebral arteries are replaced by a solitary azygos (fused) anterior cerebral artery consistent with semilobar holoprosencephaly (red arrow)

**Figure 3:**
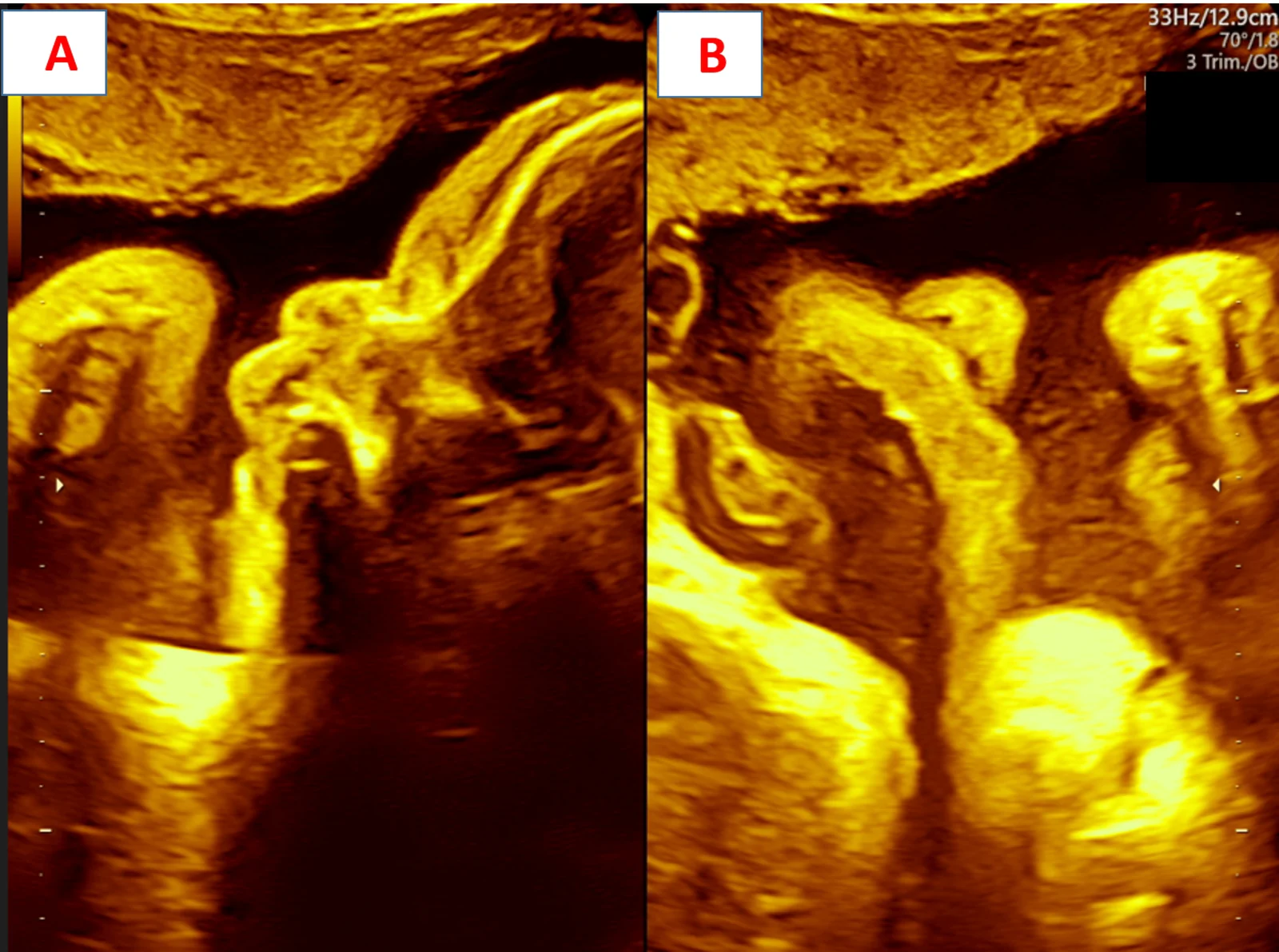
Fetal face profiling on ultrasound scan in the second trimester during the first pregnancy Fetal face profiling on ultrasound scan, panel A - midsagittal view of face showing normal nose, lip, and micrognathia, and panel B - coronal view of lower face showing normal nose, upper lip (absence of cleft)

**Figure 4:**
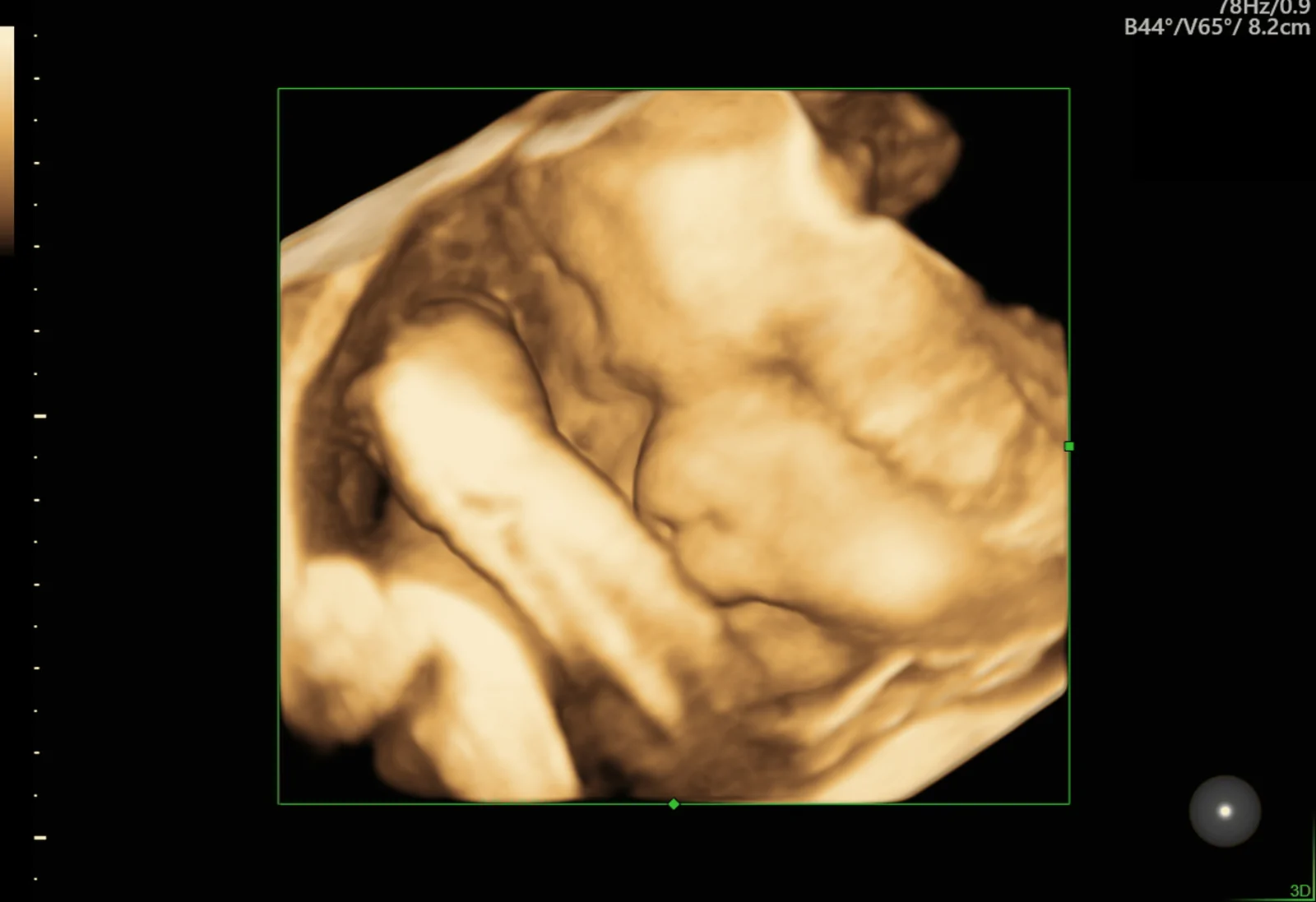
Fetal face profiling on ultrasound: two-dimensional/three-dimensional surface rendering in the first pregnancy Fetal face profiling on ultrasound: two-dimensional/ three-dimensional surface rendering shows depressed nasal bridge, normal nose, normal lips and micrognathia. The left hand (clenched fist) and forearm partially covers the face.

Genetic testing was advised but was not affordable to the family. They decided on medical termination of pregnancy, knowing the poor prognosis of the fetus. Postnatal photos of the fetus showed a tall forehead, normal nose, lips, and mouth; the limbs showed hyperextended knees and clenched fists (Figure 5).

**Figure 5:**
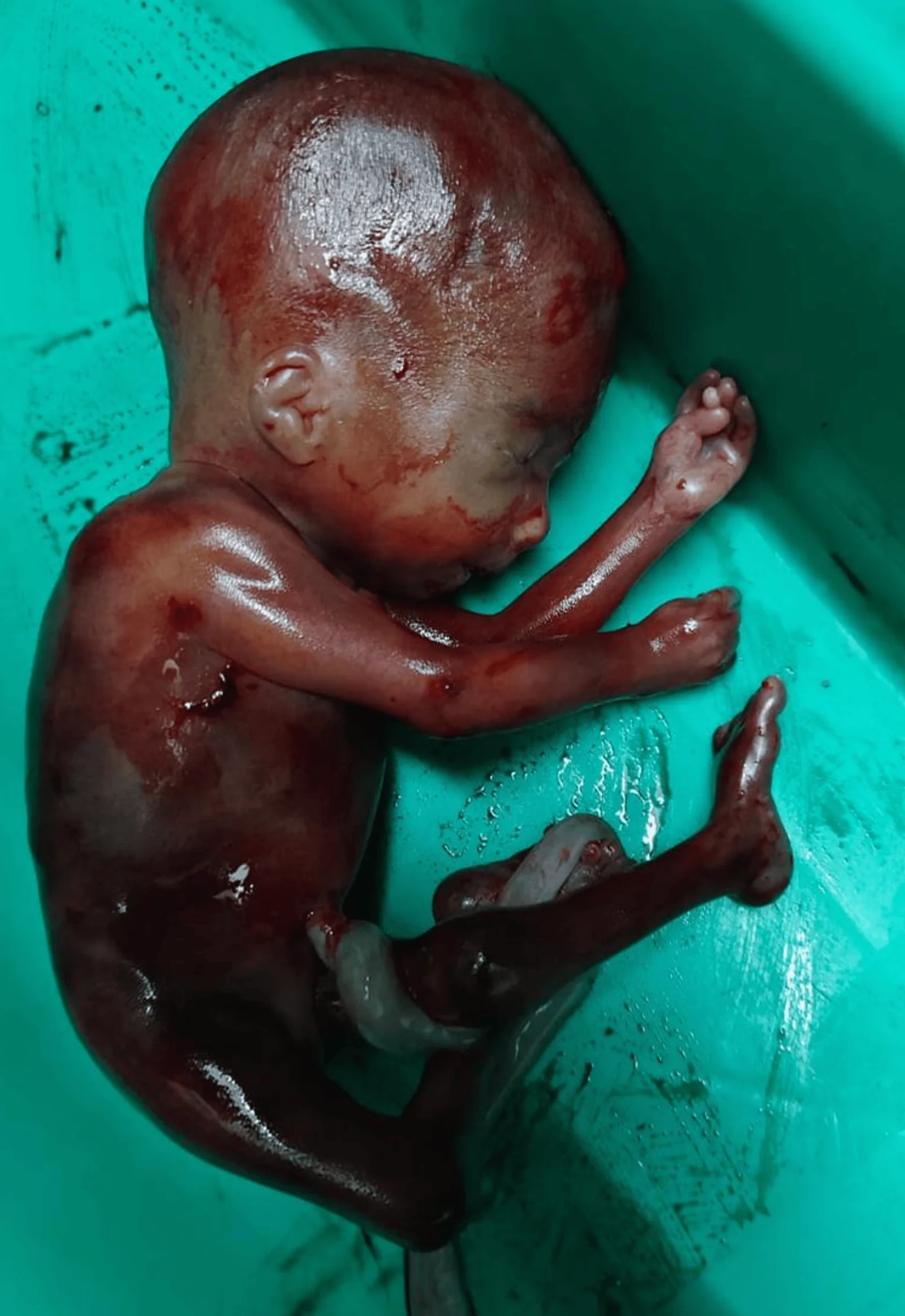
Postnatal image of the fetus delivered after the first pregnancy Postnatal image of the fetus delivered after the first pregnancy, lateral side view showing dysmorphism- tall forehead, flat nasal bridge, anteverted nares, long philtrum, jaw, clenched fists, hyperextended left knee, feet appear normal, spine is normal

After a period of three years, the lady conceived again naturally and presented to us around 20 weeks. This time, too, the fetus showed similar findings. The scan showed the following biometry: biparietal diameter 5.61 cm (23 weeks 1 day), head circumference 20.42 cm (22 weeks 4 days), occipital-frontal diameter 7.07 cm, abdominal circumference 14.82 cm (20 weeks 1 day), femur length 3.51 cm (21 weeks 1 days), humerus length 3.21 cm (20 weeks 5 days), trans-cerebellar diameter 1.56 cm (16 weeks 1 days), transverse diameter of the cisterna magna was 7.18 mm (Figures 6-8).

**Figure 6:**
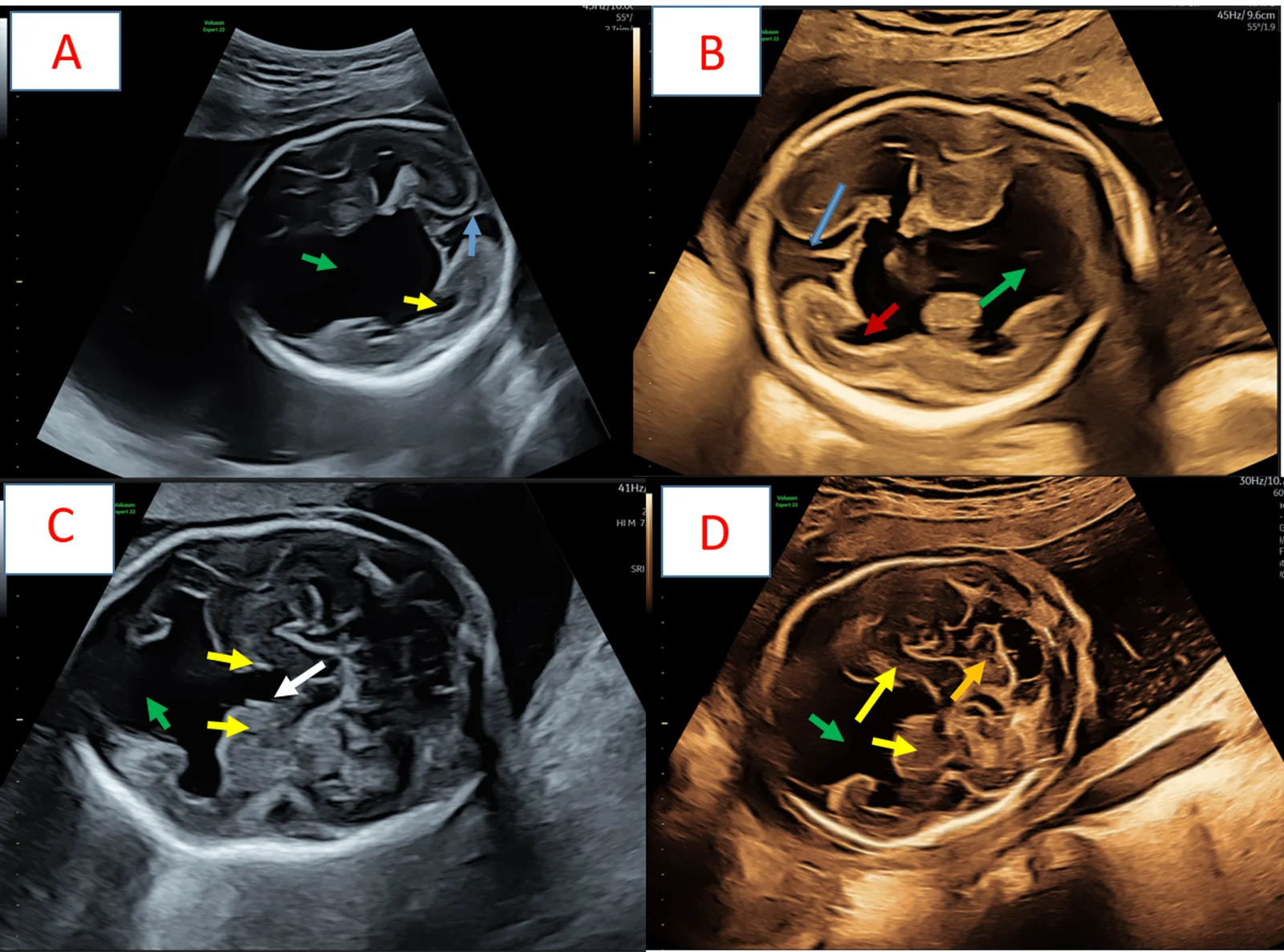
Ultrasound image of the fetal brain showing semilobar holoprosencephaly in the couple's second pregnancy Fetal ultrasound in the couple’s second pregnancy showing A) transverse section image of the fetal brain at a level above the thalamus, B) transverse plane passing at the level of the frontal horns of the lateral ventricles, C) transverse plane at the level of the partially fused thalami and, D) transverse plane passing the partially fused thalami (yellow arrows) and the cerebellum (orange arrow). The images show a mono-ventricle (green arrow) with absent falx cerebri anteriorly but present posteriorly (blue arrow) with small posterior horn (red arrow). Third ventricle (white arrow) is also seen.

**Figure 7:**
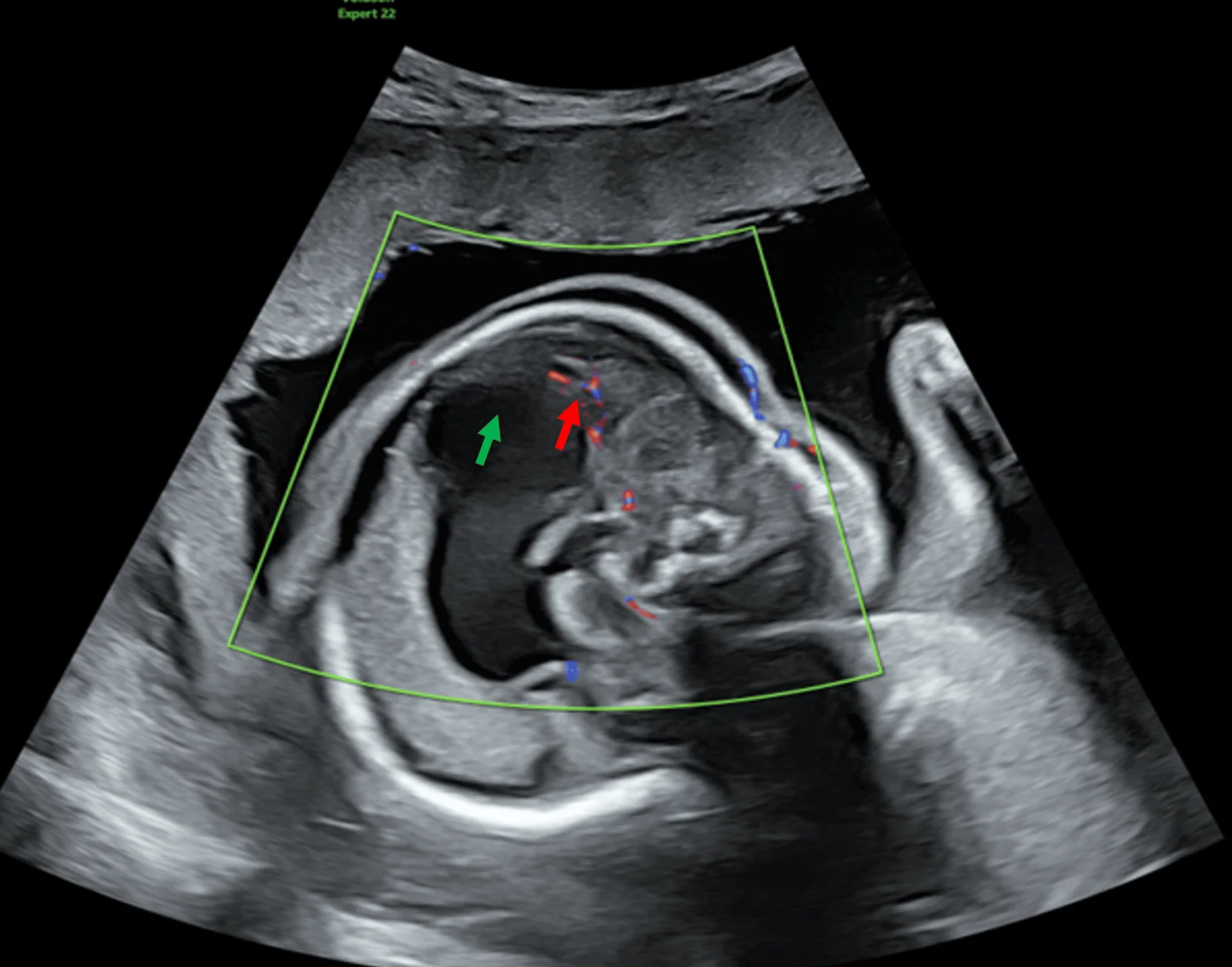
Colour Doppler ultrasound of the fetal brain in the second trimester in the second pregnancy, midsagittal plane Fetal brain ultrasound showing semilobar holoprosencephaly, in this sagittal section, one can note severe disruption of the midline architecture, lack of normal corpus callosum, and presence of a monoventricle anteriorly (green arrow). Doppler also shows the displacement of the cerebral vasculature. The anterior cerebral arteries are replaced by a solitary azygos (fused) anterior cerebral artery (red arrow) consistent with semilobar holoprosencephaly.

**Figure 8:**
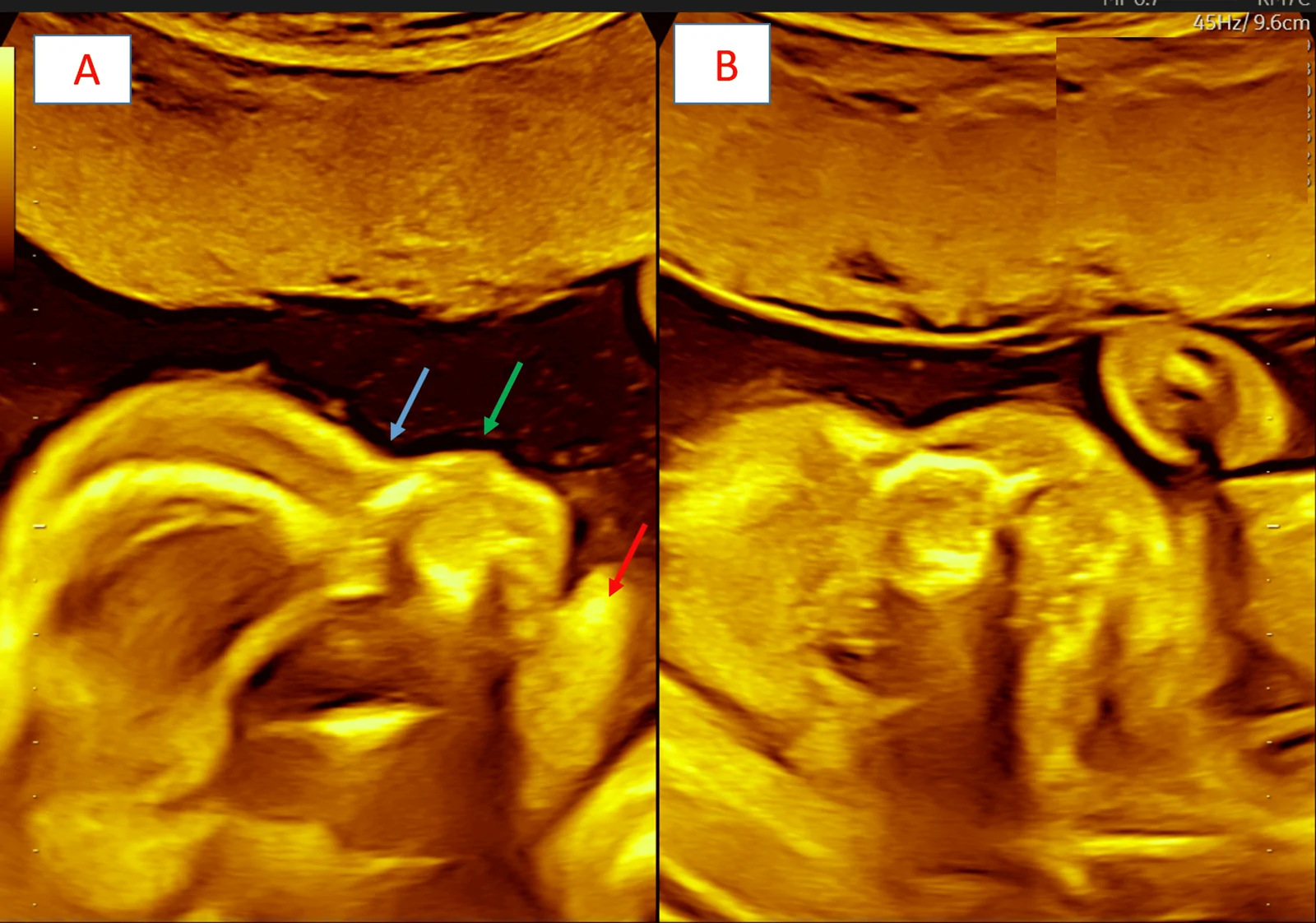
Antenatal ultrasound in the second pregnancy showing A: sagittal section and B: parasagittal section of the fetal face Sagittal and parasagittal sections of the fetal face during antenatal ultrasound in the second pregnancy showing depressed nasal bridge (blue arrow), normal nose (green arrow), micrognathia (red arrow)

The family decided to go ahead with fetal testing this time. The pregnancy was terminated, and the postnatal skin tissue was sent for DNA analysis. The fetus showed dysmorphism: tall forehead, flat nasal bridge, anteverted nares, long philtrum, and jaw (Figure 9).

**Figure 9:**
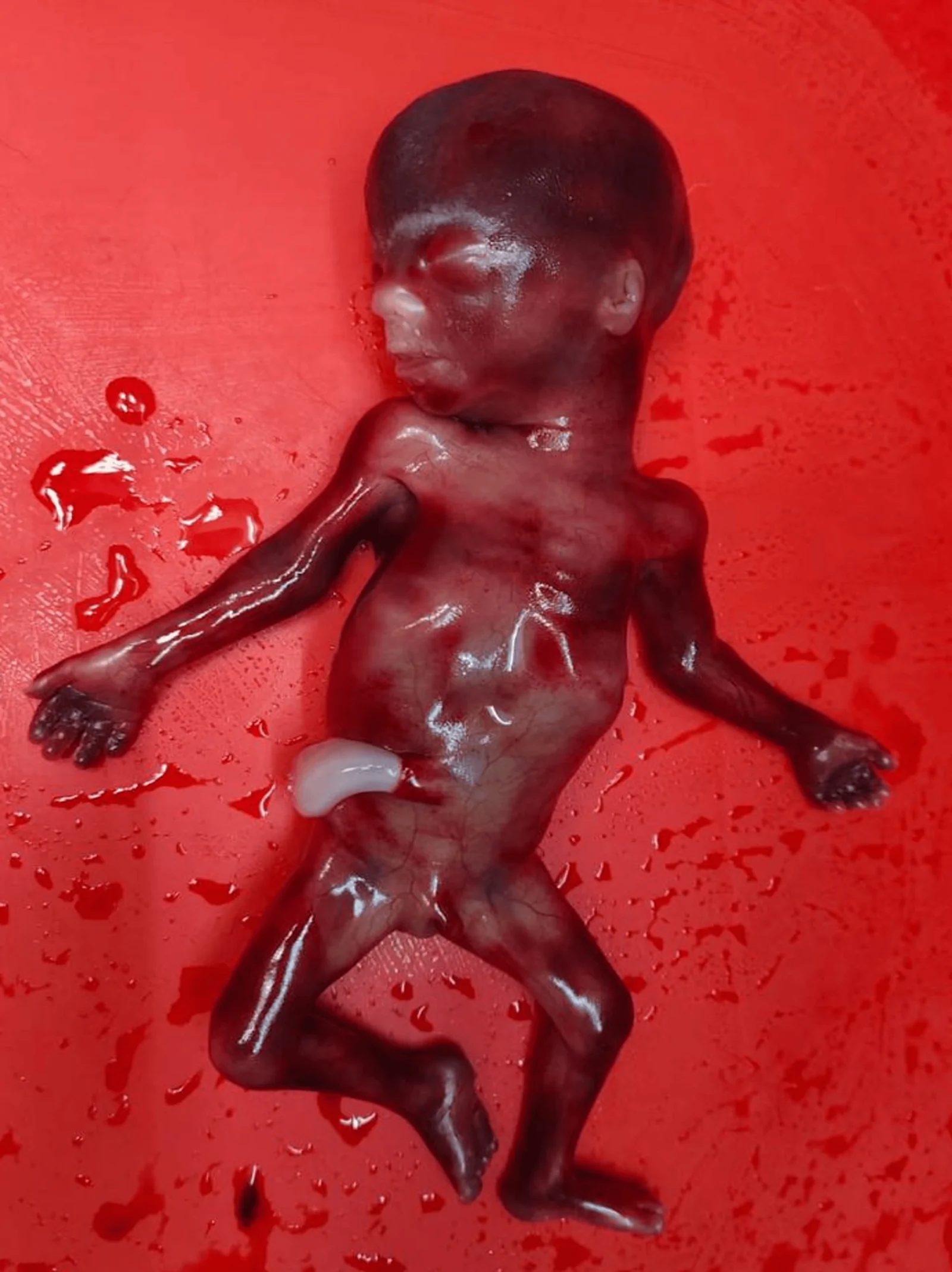
Postnatal image of the fetus delivered after the second pregnancy Postnatal image of the fetus delivered after the second pregnancy showing dysmorphism- tall forehead, flat nasal bridge, anteverted nares, long philtrum, jaw. The limbs, thorax, and abdomen were normal. No detailed autopsy could be conducted.

Both cases were definitively classified as semilobar HPE rather than alobar or lobar variants based on their distinct intermediate neuroimaging features. Alobar HPE is characterized by a complete single monoventricle with zero posterior separation and absent interhemispheric structures, whereas lobar HPE demonstrates nearly complete hemispheric and ventricular separation with a distinct cavum septum pellucidum [1,2]. In contrast, both fetuses in these pregnancies exhibited hallmark features of the semilobar category: an anterior monoventricle with fused frontal horns alongside distinctly separated or partially formed posterior and occipital horns, a midline falx and interhemispheric fissure that were absent anteriorly but present in the occipital region, and partially fused thalami. Furthermore, the presence of a solitary (azygos) anterior cerebral artery and intermediate cerebral cleavage definitively placed these findings in the semilobar spectrum, safely ruling out the more severe alobar and milder lobar forms [1,2].

The fetal samples showed the absence of Toxoplasma, rubella virus, and cytomegalovirus by polymerase chain reaction. Fetal chromosomal microarray performed on Applied Biosystems™ CytoScan™ Optima 750K chip (Thermo Fisher Scientific Inc., Waltham, MA, USA) did not reveal any significant chromosomal aneuploidies or microdeletion-duplication syndromes. The gene *BORCS8* (MEF2B) was found in a region of loss of heterozygosity arr[hg19] 19p13.2p12(13,017,875-20,684,670) hmz (around 7667 kilobases), which suggested identity by descent of this region (result of consanguinity) (Figure 10).

**Figure 10:**
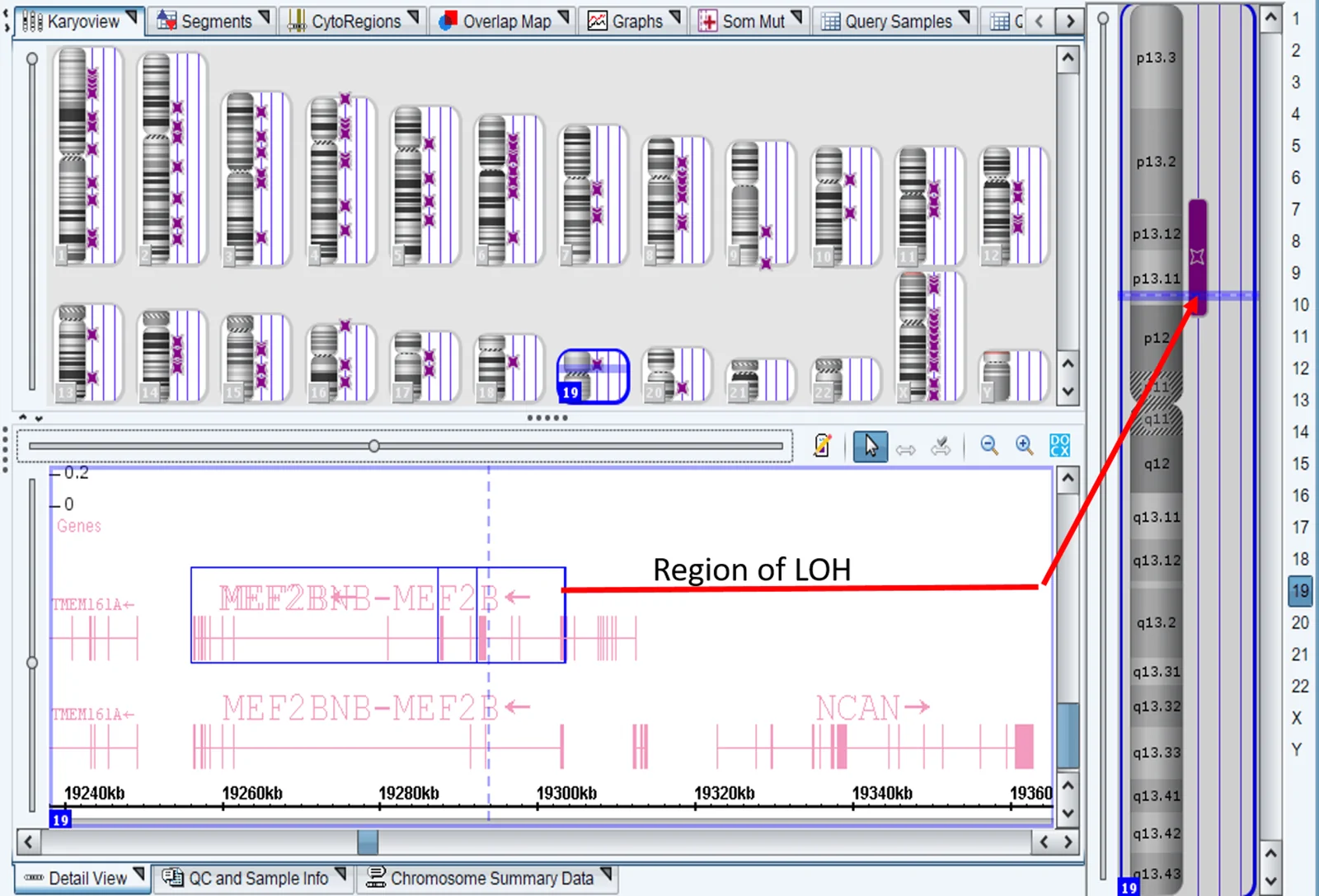
Chromosomal microarray performed on fetal DNA - virtual karyogram Chromosomal microarray on fetal DNA performed using Applied Biosystems CytoScan Optima 750K chip (Thermo Fisher Scientific Inc., Waltham, MA, USA) showing no chromosomal aneuploidies or microdeletion/duplication syndromes. The gene *BORCS8* (MEF2B) was found in a region of loss of heterozygosity on chromosome 19 (red arrow). Coordinates of the region of loss of heterozygosity (LOH) on chromosome 19 are arr[hg19] 19p13.2p12(13,017,875-20,684,670) hmz (around 7667 kilobases). This suggested identity of this region by descent (result of consanguinity).

Fetal whole exome analysis (including 20321 nuclear genes and 37 mitochondrial genes) was performed on the genomic DNA extracted from fetal tissue on the Illumina NovaSeq 6000 platform (Illumina, Inc., San Diego, CA, USA) with paired-end 2x150 chemistry. We followed the Illumina Dynamic Read Analysis for Genomics (DRAGEN) Bio-IT Platform for the identification of variants in the sample. We assembled the sequences and aligned them to reference sequences based on National Center for Biotechnology Information (NCBI) RefSeq transcripts and the human genome build (GRCh38). Clinically relevant mutations in both coding and non-coding regions were annotated using published variants in the literature and a set of disease databases: OMIM, ClinVar, Genome Aggregation Database (gnomAD), 1000Genomes database, and our internal database [5,7-9]. The genomic data were also screened for CNVs using ExomeDepth (version 1.1.10) [10]. Exome sequencing generated a total of 11.7 gigabases with 99.46% of reads aligned and 94.7% of reads having more than Q30 base-call accuracy. The mean depth was 100.91x (1x: 98.36 %; 5x: 98%; 20x: 97.63 % reads). The variant call format (VCF) file thus generated contained 61,599 variants in 14,725 genes. The VCF file was annotated using Franklin Genox software [11].

The fetus was thus identified to be homozygous for a novel pathogenic consensus splice site variant in the *BORCS8* gene (c.150+1G>T or chr19-19186892 C>A, transcript ID NM_001145784.2). The variant satisfies the following American College of Genetics and Genomics (ACMG) criteria for pathogenicity: PVS1- null variant in a gene where loss of function is a known mechanism of disease, and PM2_supporting - absent in the gnomAD database and absent in our internal database of 50000 exomes. Hence, the variant is classified as likely pathogenic by nature but could be considered a variant of uncertain significance (VUS) since HPE is not a reported phenotype (Figure 11).

**Figure 11:**
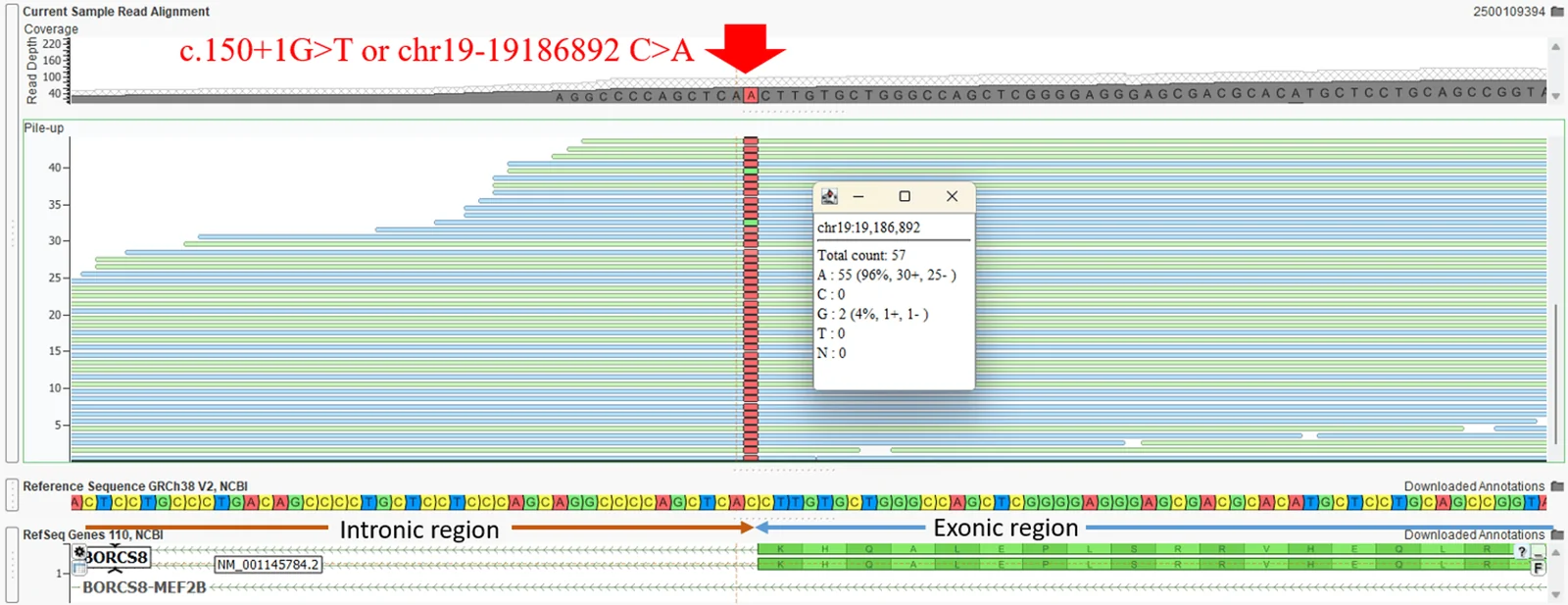
Genomic view of the binary alignment map file after exome sequencing analysis using Varseq software (Golden Helix Inc., Bozeman, MT, USA) showing a homozygous variant in the BORCS8 gene The image shows that the fetus was homozygous for a novel pathogenic consensus splice site variant in the *BORCS8* gene (c.150+1G>T or chr19-19186892 C>A, transcript ID NM_001145784.2) (red arrow). This is a substitution of adenine (A) for cytosine (C) on both chromosomes. The white box depicts the number of reads as 57x with an A nucleotide represented in 55 reads; the two reads representing a G nucleotide were ignored as aberrant reads. This is an exon-intron boundary, a consensus donor splice site.

No significant variant was seen in the following known genes for HPE: *C2CD3, CDON, CNOT1, CENPF, DHCR7, DISP1, DLL1, EMX2, FGF8, FGFR1, GLI2, GLI3, ICK, IFT80, KMT2D, PPP1R12A, PRRX1, PTCH1, RAD21, SHH, SIX3, SMC1A, STAG2, STIL, TGIF1, WDR81, *or* ZIC2. *Targeted Sanger sequencing was done for the *BORCS8* gene variant in the fetus and asymptomatic parents, which confirmed the fetus to be homozygous and the parents as carriers (heterozygous) for the same variant, confirming autosomal recessive inheritance in the family (Figure 12).

**Figure 12:**
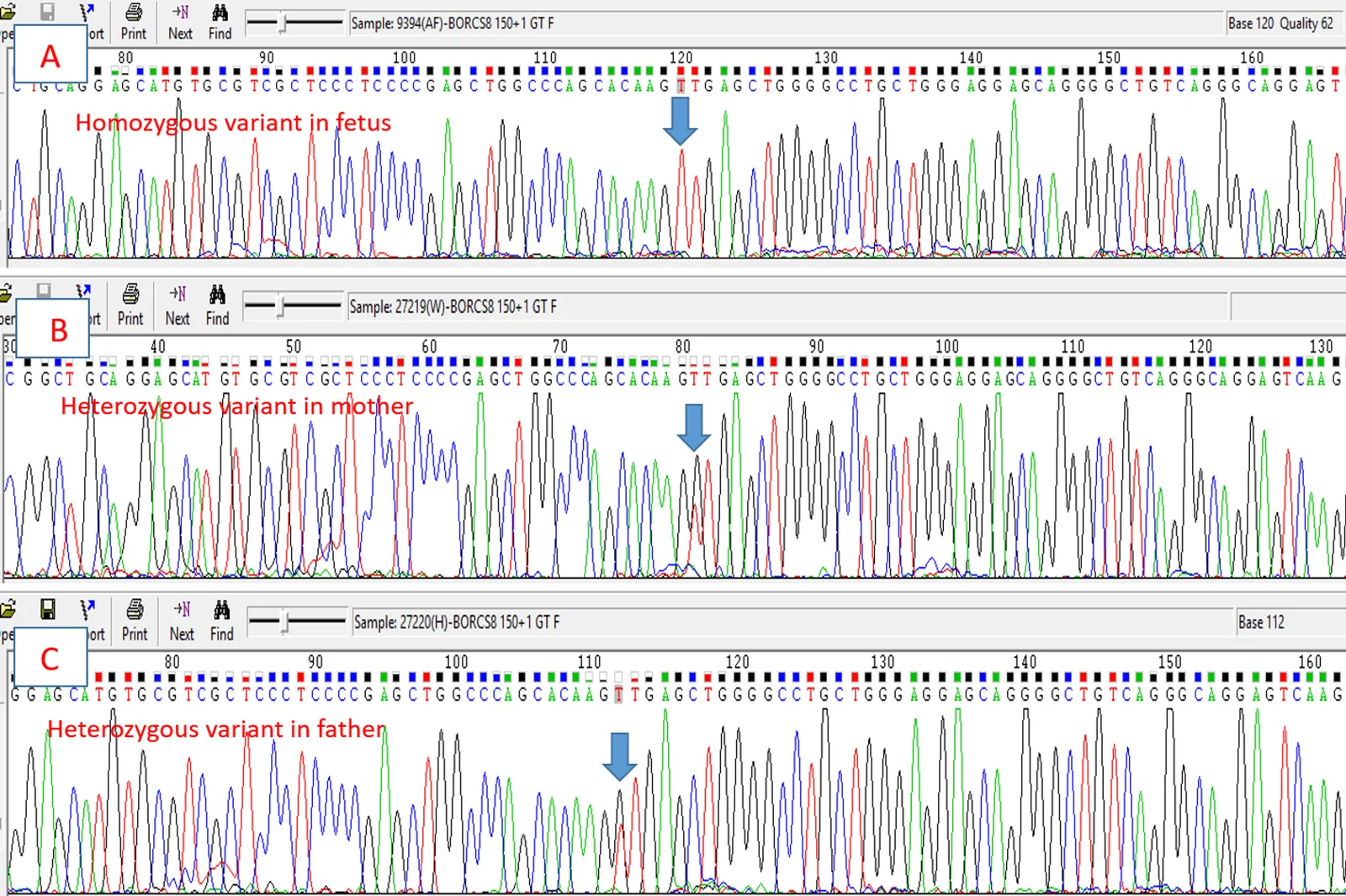
Targeted Sanger sequencing for the BORCS8 gene in the fetus and parents Targeted Sanger sequencing for the pathogenic variant in *BORCS8* gene viz c.150+1G>T (transcript ID NM_001145784.2) in the fetus (panel A) and the parents (panels B and C). The homozygous variant is seen in the fetus (panel A) (both T alleles represented by a red peak). The heterozygous variants are seen in the mother (panel B) and the father (panel C) and are represented by two peaks - a red peak representing T nucleotide and a black peak representing G nucleotide.

The family received genetic counselling, and they can now avail prenatal diagnosis and/or pre-implantation genetic diagnosis to prevent recurrences in their future progeny.

A comparison of findings between the two pregnancies of the couple is given in Table 1.

**Table 1:** Comparison of findings seen in the two pregnancies of the couple Two consecutive pregnancies of the couple leading to medical termination of pregnancy due to fetal semilobar holoprosencephaly.

Feature / Parameter	First Fetus (First Pregnancy)	Second Fetus (Second Pregnancy)
Gestation	Late second trimester (~26 weeks at anomaly scan)	Around 20 weeks
Brain Malformation	Semilobar holoprosencephaly (fused frontal horns and bodies of lateral ventricles, cleaved thalami). Absent midline falx anteriorly, present posteriorly. Rhombencephalosynapsis (cerebellum below 1st centile with absent vermis/fused hemispheres)	Semilobar holoprosencephaly (mono-ventricle, partially fused thalami). Absent falx cerebri anteriorly, present posteriorly. Small posterior horn, third ventricle present. Cerebellar/posterior fossa involvement
Facial & Limb Features	Facial: Tall forehead; normal nose, lips, and mouth. Limbs: Hyperextended knees and clenched fists	Facial: Dysmorphic features including tall forehead, flat nasal bridge, anteverted nares, long philtrum, and jaw. Limbs: Normal (thorax and abdomen also normal)
Genetic Findings	Advised, but not performed due to financial constraints (not affordable)	Postnatal skin tissue sent for DNA analysis: Microarray normal, Exome sequencing: homozygous mutation in BORCS8 gene

A protein network analysis using the Search Tool for Retrieval of Interacting Genes/Proteins (STRING) database did not show any interactions between the *BORSC8* gene and other genes causative for holoprosencephaly - *C2CD3, CDON, CNOT1, CENPF, DHCR7, DISP1, DLL1, EMX2, FGF8, FGFR1, GLI2, GLI3, ICK, IFT80, KMT2D, PPP1R12A, PRRX1, PTCH1, RAD21, SHH, SIX3, SMC1A, STAG2, STIL, TGIF1, WDR81, *or *ZIC2 * (Figure 13) [12].

**Figure 13:**
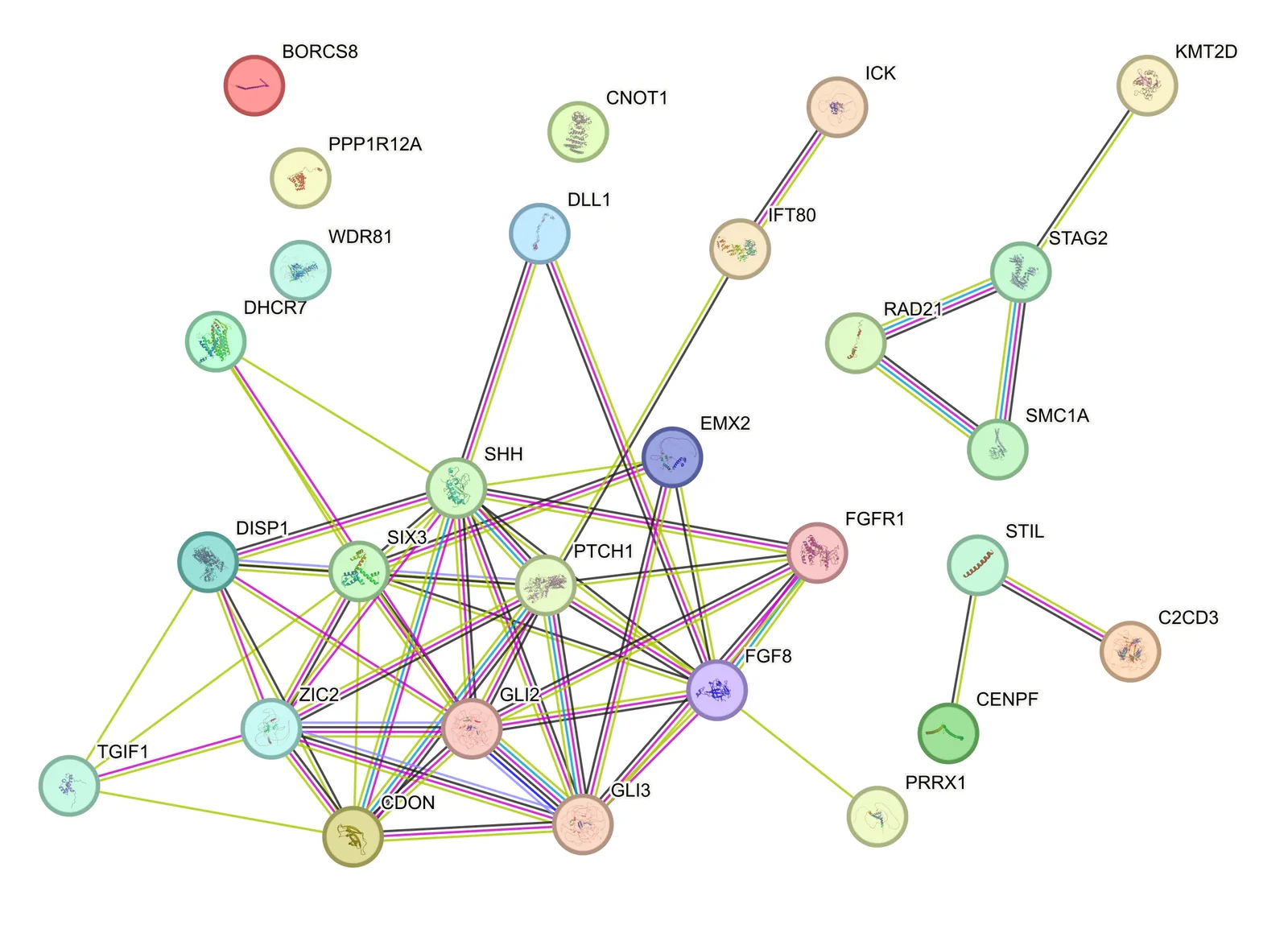
Search Tool for Retrieval of Interacting Genes/Proteins (STRING) database analysis of BORCS8 gene and other genes known to be causative for holoprosencephaly STRING database analysis did not show any interactions between the *BORSC8* gene and other genes causative for holoprosencephaly - *C2CD3, CDON, CNOT1, CENPF, DHCR7, DISP1, DLL1, EMX2, FGF8, FGFR1, GLI2, GLI3, ICK, IFT80, KMT2D, PPP1R12A, PRRX1, PTCH1, RAD21, SHH, SIX3, SMC1A, STAG2, STIL, TGIF1, WDR81, ZIC2 *[12]

## Discussion

HPE is a developmental anomaly of the brain wherein there is a failure of division of the prosencephalon (forebrain) into two hemispheres during the embryonic period. The phenotypic spectrum of HPE varies from alobar HPE (most severe) to lobar HPE (least severe). HPE is genetically heterogeneous, with several genes reported and inheritance patterns such as AD, AR, and X-linked [2]. The OMIM also lists several of these loci [5]. The genes responsible for non-syndromic forms include *SHH, ZIC2, SIX3, FGF8, DISP1, TGIF1, *and* DLL1*. The genes responsible for syndromic forms include *CNOT1, FGFR1, DHCR7, CENPF, STIL, KMT2D, PPP1R12A, RAD21, SMC1A, STAG2, CDON, PTCH1, GLI2, GLI3, EMX2, PRRX1, C2CD3, ICK, IFT80, *and* WDR81*. The AD genes include the *SHH* gene (most common gene, around 6% of cases), wherein the full spectrum of HPE is seen, *ZIC2* (upslanted eyes, large ears, short nose), *SIX3*, *FGF8* (hypogonadotrophic hypogonadism), *CNOT1* (neonatal diabetes, pancreatic insufficiency), *DISP1, FGFR1* (ectrodactyly), *TGIF1, DLL1, KMT2D* (Kabuki syndrome 1, distinctive facies, skeletal anomalies, occasional HPE), *PPP1R12A* (brain-urogenital malformation), *RAD21* [Cornelia de Lange (Cdls) [4], *CDON* (Steinfeld syndrome- radial ray defects, absent gallbladder), *PTCH1* (Gorlin syndrome), *GLI2* (Culler Jones syndrome- polydactyly, pituitary anomalies), *GLI3* (Greig syndrome/ Pallister Hall syndrome- polydactyly, hypothalamic hamartoma), *EMX2* (schizencephaly) and *PRRX1* (agnathia-otocephaly, AR forms also reported). The AR forms include *DHCR7* (Smith-Lemli-Opitz syndrome - two-to-three toe syndactyly, low serum cholesterol), *CENPF* (intestinal atresia, ocular abnormalities), *STIL* (primary microcephaly 7), *C2CD3* (orofaciodigital syndrome type 14, ciliopathy), *ICK* (endocrine-cerebrao-osteodysplasia, multisystem disease), *IFT80* (Jeune syndrome 2, asphyxiating thoracic dystrophy) and *WDR81* (CAMRQ syndrome, cerebellar ataxia, mental retardation and quadrupedalism). The X-linked dominant genes include *SMC1A* (Cdls 2) and *STAG2* (congenital heart defect, cleft lip-palate) [2]. The *BORCS8* gene is presently not included in the list of causative genes for HPE as per GeneReviews, the OMIM website, and a MEDLINE (National Library of Medicine) search [2,5,6].

BORC is made of eight distinct named proteins (labelled BORCS1 through BORCS8). It acts as a master regulator for peripheral lysosome positioning by acting on the cytosolic face of lysosomes to recruit the small GTPase Arl8. Arl8 subsequently activates plus-end microtubule motors, specifically Kinesin-1 (KIF5B) and Kinesin-3, which transport the lysosomes toward the cell periphery [3].

De Pace et al. (2023) identified biallelic variants in the *BORCS8* gene in a severe form of infantile neurodegeneration. Five children from three unrelated families presented with clinical features including global developmental delay, profound intellectual disability, axial hypotonia, limb spasticity, and muscle wasting. They also showed facial dysmorphism, optic atrophy, and brain imaging that revealed leuko-axonopathy with hypomyelination, primarily affecting the supratentorial region of the brain. Cellular transfection studies showed that while missense variants (p.Ser29Pro, p.Ser42Pro, and p.Thr66Pro) were produced at normal protein levels, they were functionally defective, failing to complex correctly with other BORC subunits. This failure disrupted the BORC complex’s ability to push the lysosomes toward the periphery of the cell. The frameshift variant (p.Asn26Trpfs51) proved to have an even more deleterious effect, showing lower expression levels and a total inability to assemble or facilitate lysosome movement. Their study concluded that these mutations represent partial or total loss-of-function alleles, making them the likely cause of the disease. To confirm this, they created a zebrafish knockout model of the *borcs8* gene, which successfully mirrored the human condition by exhibiting reduced brain and eye sizes, neuromuscular defects, and difficulty moving. This approach confirmed that *BORCS8* is vital for proper lysosomal distribution and overall neurological development in infancy/early childhood [3].

In a 2026 case study, Abdel Hamid et al. reported on a six-month-old Egyptian male who presented with the recently identified BORCS8-related neurodevelopmental disorder, caused by a homozygous p.Ser42Pro missense variant. The infant’s clinical profile began with a low birth weight and progressed into severe growth failure, affecting his weight, length, and head circumference, alongside persistent feeding difficulties and distinct craniofacial dysmorphism. Neurologically, the child exhibited profound cognitive and motor delays, generalized hypotonia, and an absence of the patellar reflex, complemented by symptoms of dystonia, seizures, clenched hands, and distal wasting in the lower limbs. Ophthalmic evaluations further identified optic atrophy and cortical visual impairment. Detailed neuroimaging revealed a complex set of brain anomalies, including mild cerebral and cerebellar atrophy, ventricular dilatation, and partial agenesis of the corpus callosum. Additionally, the imaging showed pontine hypoplasia, underdevelopment of the inferior vermis, and specific hypointensity within the globus pallidi. This comprehensive case reinforces the severe, multi-systemic impact of *BORCS8* mutations on early human development and neurological structural integrity [4].

We postulate that our case had a severe presentation since this is likely an amorphic type (complete loss of function) of variant, and to date, no patient with a homozygous amorphic variant has been described. Previously described childhood-onset cases have been homozygous hypomorphs (partial loss of function) or compound heterozygous for an amorphic variant along with a hypomorphic variant.

We compare the findings seen in our cases with findings known to be currently associated with *BORCS8* gene-related disease in Table 2.

**Table 2:** Comparison between findings in the present two fetuses in our case and findings known to be currently associated with BORCS8 gene-related disease Comparison between our case and the *BORCS8* gene-related cases in the medical literature suggests that cases with homozygous amorph or null-type variants may present as a severe presentation of semilobar holoprosencephaly in fetal life, whereas cases with hypomorphic variants or amorph-hypomorph combination can present in early post-natal life with leukoencephalopathy and neurodegeneration. Adapted from [1-4].

Category / System	Present Case Findings (Both Fetuses)	Known Features in BORCS8 Gene-Related Cases (Literature)
Presentation & Timing	Prenatal diagnosis (Second trimester: ~20 to 26 weeks) across successive consanguineous pregnancies	Infantile-onset postnatal neurodegeneration and developmental arrest
Major Brain Malformations	Semilobar holoprosencephaly (mono-ventricle, fused frontal horns/bodies, partially cleaved thalami). Rhombencephalosynapsis (absent cerebellar vermis, fused cerebellar hemispheres). Absent midline falx anteriorly, present posteriorly	Leuko-axonopathy with hypomyelination/leukodystrophy. Supratentorial neurodegenerative features. Cerebral atrophy, small pons, inferior cerebellar vermis. Microcephaly (Note: Classic forebrain cleavage defects like holoprosencephaly and rhombencephalosynapsis are not typical core hallmarks of BORCS8)
Ophthalmological Findings	Not detailed/normal structural appearance prenatally	Optic atrophy (consistently documented across affected individuals)
Facial Dysmorphism	Tall forehead; flat nasal bridge; anteverted nares. Long philtrum and jaw	No characteristic dysmorphic facial features described
Musculoskeletal & Limb Features	Variable features observed including hyperextended knees, clenched fists, or normal limbs/thorax/abdomen	Early hypotonia progressing to limb spasticity. Muscle weakness, wasting, and atrophy. Progressive scoliosis
Neurodevelopment / Clinical Course	Medical termination of pregnancy elected in both instances due to poor prognosis	Global developmental delay and severe-to-profound intellectual disability. Seizures/epilepsy. Feeding difficulties/failure to thrive
Genetics & Inheritance	Third-degree consanguineous parents with recurrence; DNA analysis performed on second fetus, homozygous likely amorph variant in the BORCS8 gene identified, autosomal recessive inheritance	Autosomal recessive inheritance (biallelic loss-of-function variants: homozygous or compound heterozygous), likely to be homozygous hypomorphs or amorph-hypomorph combination in the BORCS8 gene

We also postulate the following mechanisms by which a pathogenic variant in the *BORCS8* gene could lead to HPE. The BORCS8 protein is a critical component of the BORC complex, which acts as a master regulator of lysosome and endosome positioning [13]. BORC recruits the small GTPase ARL8 protein, which in turn links lysosomes to kinesin motor proteins. This machinery drives the anterograde (outward) transport of lysosomes and late endosomes from the center of the cell toward the periphery (the plasma membrane or the tips of axons) [14]. The first mechanism postulated is the disruption of Sonic Hedgehog (SHH) secretion. The SHH pathway is the primary driver of midline patterning. SHH is lipid-modified and highly hydrophobic, relying on exocytosis via multivesicular bodies (MVBs) to be secreted from the notochord/prechordal plate [15]. If BORCS8 is lost, the cell cannot mobilize the SHH-containing vesicles to the cell surface. The SHH signal stays trapped in the center of the cell, leading to a localized deficiency of SHH at the midline. The second mechanism could be impaired axonal outgrowth and neuronal migration. Semilobar HPE involves not just a lack of separation of the brain hemispheres but also includes significant cortical and axonal abnormalities [15,16]. Developing neurons require the anterograde transport of lysosomes to facilitate the expansion of the plasma membrane at the growth cone. Without BORC-mediated transport, neurons may fail to migrate correctly or extend axons across the midline (like the corpus callosum), contributing to the structural fusion seen in HPE [16]. The third mechanism contributing could be defective ciliogenesis. Many HPE-related genes are associated with the primary cilium, which acts as an antenna for SHH signaling [17]. Endosomal trafficking is vital for assembling the ciliary membrane. A failure in BORC-dependent trafficking could lead to dysfunctional cilia, rendering the forebrain "deaf" to the SHH signals that are present. The homozygous loss of BORCS8 likely creates a "traffic jam" of essential signaling vesicles [17]. 

If confirmed, this would categorize the *BORCS8* gene as a neurodevelopmental "trafficking" gene. Since semilobar HPE is a mid-to-severe phenotype, it suggests that the BORC complex is indispensable for the SHH signaling required during the first three to four weeks of human gestation. 

The limitations of our study are that a detailed autopsy was lacking for both fetuses, and that RNA or protein studies could not be performed for functional characterisation of the variant in the *BORCS8* gene.

## Conclusions

Thus, we describe a novel genetic finding in a family with recurrent fetal semi-lobar holoprosencephaly, namely, homozygosity for a likely amorphic variant (consensus splice-site variant) in the *BORCS8* gene, inherited in an autosomal recessive manner. We also postulate that mechanisms leading to HPE in this syndrome could be defective SHH secretion, defective axonal outgrowth, and defective ciliogenesis. Thus, this case may expand the known phenotypic spectrum of the *BORCS8* gene (from cerebral atrophy in hypomorphic cases or amorphic-hypomorphic cases to HPE in homozygous amorphic cases). The *BORCS8* gene may represent a candidate HPE gene, and additional independent case reports are required to formally include it on HPE diagnostic gene panels. This report can thus be helpful in the future for similar cases for their genetic counseling and prevention using prenatal diagnosis and/or pre-implantation genetic diagnosis. This is only the third published paper on the *BORCS8* gene-related disease as per the PubMed database (MEDLINE).
